# Quality problems of clinical trials in China: evidence from quality related studies

**DOI:** 10.1186/s13063-022-06281-1

**Published:** 2022-04-23

**Authors:** Jin Fan, Xiaobo Liu, Yuxi Li, Haisha Xia, Rong Yang, Juan Li, Yonggang Zhang

**Affiliations:** 1 Department of Periodical Press, National Clinical Research Center for Geriatrics, West China Hospital, Sichuan University, Chengdu, China; 2 School of Health Preservation and Rehabilitation, Chengdu University of Traditional Chinese Medicine, Chengdu, Sichuan China; 3 Chinese Evidence-based Medicine Center, West China Hospital of Sichuan University, Chengdu, Sichuan China; 4 Nursing Key Laboratory of Sichuan Province, Chengdu, China

**Keywords:** China, Clinical trials, Quality control, Systematic review

## Abstract

**Background:**

Recently, the quality of clinical trials conducted in China has made considerable progress. However, clinical trials conducted in China still fall below the global average standard. The aim of this systematic review was to assess studies that investigated the quality of clinical trials conducted in China, summarize the issues, and provide suggestions for conducting high-quality clinical trials in China.

**Methods:**

We comprehensively searched studies that investigated the quality of clinical trials conducted in China in the following databases from inception to December 1, 2021: National Knowledge Infrastructure, the Chinese Science and Technology Periodical Database, WanFang Data, China Biology Medicine, PubMed, and Embase. We then analyzed the issues in clinical trial registration, ethics review, implementation, and reporting. SPSS 25.0 software was used for data analysis. The data synthesis was conducted using summary statistics and a narrative format.

**Results:**

A total of 90 studies were analyzed, there were 50 studies with 0–5 citation counts (55.56%), 18 studies with 5–10 citation counts (20%), 9 studies with 10–15 citation counts (10%), and 13 studies with more than 15 citation counts (14.44%). Eight (8.89%) studies were conducted by a supervision department, 38 (42.22%) by organizations with *GCP* qualification, and 44 (48.89%) by third parties. Additionally, there were some problems in the ethical review process of clinical trials, clinical trial registration process, clinical trial implementation process, and clinical trial reporting process.

**Conclusions:**

The current study shows that the quality problems of clinical trials in China still exist. The reported problems are related to the process of clinical trials, including ethical review, registration, implementation, reporting. Due to the limited quantity and quality of included studies, the conclusions of this study need to be verified by high-quality studies.

**Review registration:**

Not registerated in  PROSPERO.

**Supplementary Information:**

The online version contains supplementary material available at 10.1186/s13063-022-06281-1.

## Background

Clinical trials are studies based on population, human body, or samples, such as tissues and fluids [1]. Evidence from clinical trials provides a meaningful reference for doctors and policymakers in health care [2]. With the rapid development of medicine in China, the quality of clinical trials conducted in China has made considerable progress. However, the quality were still needed to improve [3, 4].

High-quality clinical trials play an irreplaceable role in clinical decisions. However, the global quality of clinical trials is facing significant challenges. Transparency and quality control during the entire clinical trial process are the most important strategies to improve this situation [5]. On July 1, 2020, the China Food and Drug Administration (CFDA) and National Health Commission published a revised *Good Clinical Practice (GCP)* claiming “Criterion for the quality control of clinical trials of drugs is the quality standard for the whole process of drug clinical trials, including designing, organizing and implementing, supervising, inspecting, recording, analyzing, summarizing, and reporting.” Registering protocols, ensuring transparency, and reporting results accurately can improve the quality of clinical trials. Although clinical trials in China have improved through optimizing research design, strictly reviewing protocols, improving researchers’ ability, and enhancing quality supervision, quality problems still exist. Based on this situation, the aim of this study is to search studies related to the quality of clinical trials in China, systematically review the current status, and summarize the existing problems to provide a reference for researchers.

## Methods

### Protocol and registration

The systematic review was not registered in PROSPERO. This systematic review was reported according to the Preferred Reporting Items for Systematic reviews and Meta-Analysis 2020 (PRISMA 2020) statement guidelines [6]. A completed PRISMA checklist was available in supplementary material 1.

### Inclusion and exclusion criteria

This systematic review included reviews that evaluated problems of domestic clinical trials. The primary outcome was the problem reported in any part of the clinical trial process. Secondary outcomes were the frequency and composition ratio. Additionally, studies were excluded if they met any of the following criteria: no problems reported, duplicate publications, confined to a particular field or special drug, and related to a medical device.

### Search strategy

Two independent reviewers (YX-L and HS-X) systematically searched the following databases from inception to December 1, 2021: China National Knowledge Infrastructure, the Chinese Science and Technology Periodical Database, WanFang, China Biology Medicine, PubMed, and Embase. The searched terms were quality, status, situation, issue, deficiency, trials, China, and Chinese. The full search strategies which tailored according to the characteristic of the above databases were listed in supplementary material 2. We collected studies that reported problems from all aspects of clinical trials in China. In addition, we reviewed the references of the included studies to obtain relevant studies. Simultaneously, we searched grey literature and the reference lists of identified studies.

### Study selection

All retrieved studies were imported into Endnote (X9) software, and then, duplicated studies were removed. Two reviewers (J-F and XB-L) independently screened the titles and abstracts to identify relevant studies in accordance with the inclusion and exclusion criteria. Subsequently, two reviewers (J-F and XB-L) downloaded the full-text of all possibly relevant studies for further assessment. Then, two reviewers (J-F and XB-L) cross-checked the included studies, and a third reviewer (YG-Z) was involved in case of disagreement.

### Data extraction

A standardized data extraction form was designed in advance. After identifying all eligible studies, two authors (J-F and XB-L) independently extracted the data according to the data extraction form. The extracted data included (1) basic information (title, first author, published journal, year, and others) and (2) types of included studies, reported problems, specific stage the problem was related to, the criteria used to evaluate the problems, and suggestions for improvement. Then, the two reviewers (J-F and XB-L) analyzed the data. Two reviewers (J-F and XB-L) resolved all discrepancies through team discussion.

### Statistical analysis

The level of agreement between reviewers was determined by the Kappa value using SPSS 25.0 software package as follows: fair agreement (0.40–0.59), good agreement (0.60–0.74), and excellent agreement (0.75 or more). We qualitatively summarized the primary outcome data. The enumeration data were described by the frequency and composition ratio. The results were summarized via tabulation.

## Results

### Included studies and characteristics

**Fig. 1:**
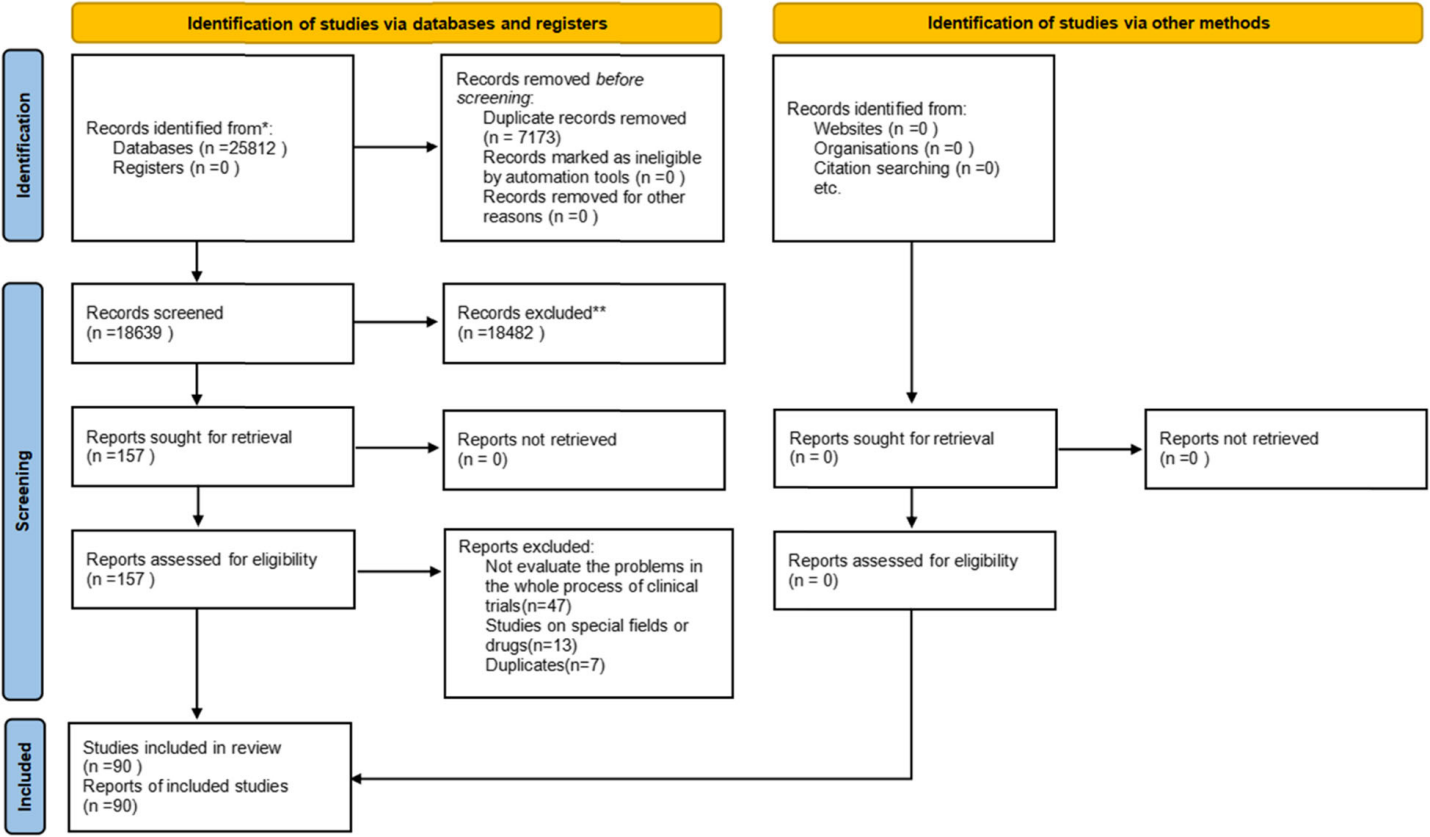
The PRISMA flow diagram for the identification of studies

We retrieved a total of 25,812 articles, of which 7173 were duplicates and thus removed. After reviewing titles and abstracts, 157 articles were retained for full-text review. After evaluating full texts, we finally included 90 [7–96] articles, which were published in 58 domestic and foreign journals. The included studies were published from 2000 to 2021. Seventy-one [7–19, 21–24, 28–31, 34, 35, 39–48, 50–52, 54–57, 60–65, 69–74, 76–89, 92–96] were full-text Chinese with English abstract studies; 19 [20, 25–27, 32, 33, 36–38, 49, 53, 58, 59, 66–68, 75, 90, 91] were full-text Chinese studies. The Kappa statistic of 0.82, 0.78 reflected excellent agreement between two reviewers for selecting studies and extracting data. The PRISMA flow diagram of the literature search is presented in Fig. 1. The full list of all 90 papers is showed in supplementary material 3.

Table 1 shows the main characteristics of the included studies. There were 50 studies with 0–5 citation counts (55.56%), 18 studies with 5–10 citation counts (20%), 9 studies with 10–15 citation counts (10%), and 13 studies with more than 15 citation counts (14.44%). Eight (8.89%) studies were conducted by a supervision department, 38 (42.22%) by organizations with *GCP* qualification, and 44 (48.89%) by third parties.

**Table 1:** Basic characteristics of included studies

Type	Clause	Number (article)	Rate (%)
Citation frequency	0–5	50	55.56
	5–10	18	20
	10–15	9	10
	> 15	13	14.44
Research resource	Supervision department	8	8.89
	Organizations with *GCP* qualification	38	42.22
	Third parties	44	48.89

### Problems in clinical trials

#### Problems in the ethical review process of clinical trials

*GCP* guidelines require that medical institutions conducting clinical trials should establish an ethics committee. The ethics committee, an organization to review ethical issues and supervise clinical trials, is established based on relevant domestic and international laws and regulations. The ethics committee is responsible for ensuring the dignity, rights, safety, and health of subjects and monitoring the conduction of clinical trials in accordance with ethical principles. In recent years, increasing clinical trials have been conducted in China, and ethical review plays an important role in the implementation of clinical trials. Nevertheless, there were many problems in the ethical review process of clinical trials in China. We included 14 [7, 11, 13, 17, 19–22, 24–27, 29, 30] studies which mentioned problems in the ethical review process of clinical trials (Table 2).

**Table 2:** Problems in the ethical review process of clinical trials

Subject	Number	Item
Ethics committee configuration	1	Unreasonable number and composition of the ethics committee [11, 13, 20, 24, 26, 30]
	2	Inadequate capacity of ethics committee members [7, 17, 26, 27, 29, 30]
Implementation	3	No review standards and norms [22, 26]
	4	Incomplete review and nonstandard records [21]
	5	Nonstandard documentation and qualification management [19]
	6	Ignoring follow-up reviews and ethical acceptance check [22, 27]
	7	No rigorous program review [25]
	8	Inadequate consideration of ethical issues (such as subsidy for participants, wash-out time, procedures for reporting serious adverse events) [25]
	9	Insufficient ethical consciousness of researchers [22]

#### Problems in the registration process of clinical trials

The International Committee of Medical Journal Editors (ICMJE) requires that all clinical trials must be internationally registered before publication. Otherwise, the results of the trials cannot be published [97]. In 2004, China established a Chinese clinical trial registration center in West China Hospital of Sichuan University based on the World Health Organization International Clinical Trials Registration Platform [98], which accepted the worldwide registration of clinical trials. Although most clinical trials conducted in China were registered, there were some problems with the registration process. We included 7 [9, 10, 12, 14–16, 18] studies which mentioned problems in the clinical trial registration process, and the results are showed in Table 3.

**Table 3:** Problems in the registration process of clinical trials

Subject	Number	Item
Researcher	1	Weak registration awareness [14]
	2	Unregistered protocol before implementation [14, 16]
Research	3	Non-standard or incomplete research protocol [9, 10, 12, 18]
	4	Lack of normative data management system [15]
	5	No claims of sharing raw data and superabundant registration [15]

#### Problems in the implementation process of clinical trials

The authenticity, reliability, and integrity of clinical trials are critical for determining the credibility of clinical trial results. Therefore, strengthening the standardized management of clinical trials is significant. Moreover, the evidence from high-quality clinical trials is used to evaluate new clinical interventions. Therefore, the quality of clinical trials directly affects the health of patients. In 2015, the CFDA reviewed the clinical data of 1622 projects, and the results showed that the quality of clinical trials conducted in China needed to be improved. At present, there were still several problems with the clinical trial implementation process regarding informed consent, protocol execution, quality control, drug management, data recording, adverse event management, biological sample handling, clinical research coordinators, and clinical trial contracts. Finally, we included 54 [8, 19, 23–25, 28, 31–61, 63, 64, 66, 69, 70, 74–81, 84–87] studies which mentioned problems in the clinical trial implementation process, and the results are showed in Table 4.

**Table 4:** Problems in the implementation process of clinical trials

Informed consent		
Subject	Number	Item
Design	1	Templated content [58, 84]
	2	Insufficient information and incorrect version [38, 40, 54, 58, 69, 76, 86]
	3	Incomprehensive description of insurance and compensation [8, 40, 84]
	4	Unreviewed and unapproved by the ethics committee [70, 86]
Implementation	5	Nonstandard informed consent signing and writing [24, 38, 54, 63, 85, 86]
	6	Lack of contact information and signing date [32, 80, 84]
	7	Deficient notification [58]
	8	Selective or induced notification [28, 38, 58, 70, 85, 86]
	9	Inappropriate place of notification [85]
	10	Unprovided copy of the informed consent to the patient [32, 41, 54, 84]
	11	Absence of informed consent signed by screening subjects [80]
	12	Informed consent process is not reflected in the original medical record [32]
Protocol execution		
Subject	Number	Item
Implementation	1	Unstrict execution of inclusion and exclusion criteria [19, 25, 32, 46, 63, 64, 75, 77, 78, 80, 81]
	2	Administration of drugs not in accordance with dosage specified in the protocol [77, 80]
	3	Premature or delayed assessment [32, 46, 57, 60, 75, 80]
	4	Time-overlapping between informed consent, screening, enrolment, and administration of drugs [24]
Record	5	Taking unspecified drugs and not recorded or not recorded on time [24]
	6	Incomplete documentation of clinical trials process [39]
	7	The signing time of the corresponding task assignment form, training record form, and the protocol signing page did not conform to the actual situation when the researchers change or the task assignment changes [24]
Quality control		
Subject	Number	Item
Inspectors	1	Inadequate execution of tertiary quality controls [44, 50, 61, 87]
	2	Insufficient competence and responsibility of the inspectors [36, 49, 56, 87]
Drug management		
Subject	Number	Item
Implementation	1	Nonstandard management in experimental drugs receipting, distribution, storage, recycling, and destruction [24, 42, 53, 59]
	2	No standard for the label of experimental drugs [59]
	3	Nonstandard records of drug administration [63, 79]
	4	The information of drug administration forms does not match with the records in original medical reports and case report forms [24, 79]
	5	The records do not present the actual dosage of drugs [32]
	6	The drug dosage and specifications recorded in drug release form are not matched with the reality [32, 80]
	7	Researchers lack knowledge on quality management practices in drug clinical trials [33, 47, 80]
Data record		
Subject	Number	Item
Implementation	1	Nonstandard record and revision of case report form [25]
	2	Missing or incomplete records of drug combination [32, 57]
	3	Inconsistent data records with primary material [32, 51, 77, 80]
	4	Data is not recorded in the medical records or not recorded in time [25]
	5	Data is untraceable, irregular, omitted, and concealed records of adverse events in trials [32, 80]
	6	Incomplete records of the reports [32, 74]
Adverse events management		
Subject	Number	Item
Judgment	1	Absence of risk prediction, prevention mechanism, and treatment for serious adverse events in the study protocol [55]
	2	Confuse clinical trials with clinical treatment [35]
	3	Absence of report on adverse events [55]
	4	Delayed time in submission of report on adverse events [23, 34, 35, 55]
	5	Misjudgment of abnormal inspection results [35]
	6	No dynamic observation on inspection results [35]
	7	Misjudgment of the causal relationship between adverse events and experimental drugs [35]
Record	8	Nonstandard report of adverse events, including deferred report and improper writing of report form [34, 55]
	9	Incomplete original records [35]
	10	Incomplete receipt collection [34]
	11	Special circumstances are not noted [34]
Biological sample handling		
Subject	Number	Item
Implementation	1	Inadequate collection, storage, transportation, and handover records of biological sample [24, 77]
Record	2	Disorder timeline of records in sample collection, inspection, and audit process [24]
Clinical research coordinators		
Subject	Number	Item
Coordinator	1	Uneven ability of clinical research coordinators [48, 66]
System	2	Imperfect construction of management organization system [37, 48]
	3	Incomplete training and assessment mechanism [37, 66]
	4	Lacking unified management system [37, 66]
Clinical trials contract		
Subject	Number	Item
Contract	1	No standards and principles for reviewing clinical trials protocols [45]
	2	No legal professional participant in review of clinical trials contract [31]
	3	Unclear injury compensation liability of participants [31, 43, 45, 79]
	4	Unreasonable clauses involving termination, confidentiality, and intellectual property ownership [31]
	5	Low purchase rate of clinical trials insurance cause the rights and interests of subjects and researchers cannot be fully protected [52]
	6	Trials contracts contain missing clauses, including description of clinical trials costs, contract signatory, and responsibilities of all parties [31]

#### Problems in the reporting process of clinical trials

The reporting of clinical trials is a summary of the design and implementation process written in accordance with reporting guidelines. The reports enable readers to understand the entire trial process and interpret the results. Additionally, the reporting of clinical trials is imperative for evaluating the effectiveness and safety of the intervention. The implementation of reporting guidelines of clinical trials is an important process to ensure the quality of reporting. In our study, we identified 19 [62, 64, 65, 67, 68, 71–73, 82, 83, 88–96] studies related to several problems in the reporting process of clinical trials (Table 5).

**Table 5:** Problems in the reporting process of clinical trials

Section	Number	Item
Abstract	1	Insufficient report of title, trials design, allocation concealment method, and trials registration [82, 88]
Methods	2	Insufficient description of random allocation sequence, allocation concealment, blinding, data analysis, and the processing of missing data in method section [62, 64, 65, 67, 71–73, 83, 88–96]
Results	3	Low report rate of subject’s flow chart and compliance [62, 73, 88]
Discussion	4	Insufficient analysis of the causes of major adverse events [71]
	5	Few studies mention and analyze the limitations of trials in detail [68]
Other	6	Studies published in Chinese have low rate of registration and incomplete outcome [65]

## Discussion

Recently, evidence-based medicine helps to standardize the classification of health care outcome research. Randomized control trials (RCTs) are standard trials designed to verify the efficacy of a certain intervention. The results of high-quality RCTs provide the most reliable evidence regarding the efficacy of healthcare interventions [99–102]. In general, *GCP* assures that the data and results are credible and protect the rights and integrity of subjects. The country issued a series of laws and regulations related to the registration and approval of new drugs in 1999, and *GCP* (issued in 2003) has become a formal implementation requirement of clinical trials of drug in China. However, in China, problems regarding the design, implementation, and reporting of clinical trials still exist. Understanding current clinical trial issues is important for the development of high-quality clinical trials in the future. Therefore, we conducted the current systematic review to summarize the problems in the entire trial process and provide suggestions for high-quality clinical trials. A total of 90 studies related to the quality of clinical trials conducted in China were included. Among them, 42.22% were conducted and summarized by clinical trial institutions (*GCP* centers) or regulatory agencies based on completed clinical trials, thereby reflecting the actual problems in clinical trials of China. The results showed that the current problems in clinical trials of China mainly involved four sections, including ethics review, clinical trial registration, implementation, and reporting.

Before the commence of clinical trials, the investigators should carry out ethical review to ensure the rights and interests of subjects [103]. Our study identified various issues regarding ethics committees in China, including an insufficient numbers, structure, or capacity of members and inadequate implementation, supervision, acceptance, and audit. Considering this situation, an ethics committee training institution should be organized to provide specialized training and academic networks for the ethics committee members. Second, the regulatory process of the ethics review committee should be improved, the extent of supervision by the ethics review committee should be strengthened, and the ability of ethics review members should be enhanced. Finally, the internal members of the ethics committee should clarify the responsibility of all parties and strengthen communication and cooperation to improve the overall ability.

Generally, the results of clinical trials and levels of evidence are helpful for clinical decision-making. However, low-quality RCTs usually provide poor-quality evidence, which might mislead clinicians. For instance, RCTs with inappropriate allocation concealment tend to exaggerate treatment effects [104, 105]. Clinical trial registration is an important measure to improve the transparency of clinical trials. Through registration of clinical trial protocols, publication bias and duplicate research can be reduced [106]. Moreover, reporting can increase the reproductivity of the research process and credibility of results. Our results showed that, in Chinese clinical trials, unregistered and post-registered phenomenon were common. Therefore, clinical investigators need to understand the importance of clinical trial registration and register their clinical protocols in advance at relevant registries (e.g., http://www.chictr.org.cn/index.aspx, https://clinicaltrials.gov/, https://www.isrctn.com/, etc.).

Clinical trials are used to determine the efficacy and safety of new interventions. Problems in the implementation of clinical trials affect the quality of clinical trials and reliability of results. This study identified several problems in the implementation of clinical trials, such as processes related to informed consent, program implementation, quality control, drug management, data recording, adverse event management, and biological sample handling. The poor quality of clinical trials is mainly due to the inadequate design and implementation of the trial protocol. International experts reveal that the impact of clinical trial design on the quality of clinical trials is even more important than the quality management system. To ensure the scientific integrity of trial protocols, the experienced methodologists and statisticians should participate in clinical trial design and statistical analysis. Additionally, clinical investigators should join in developing trial protocols and proposing timely amendments to any issues. During the implementation of clinical trials, strictly adhering to trial protocols is a prerequisite for ensuring the quality of clinical trials. However, some investigators may violate the protocol, which may induce bias in clinical trials. Clinical trial institutions should improve the quality control system and establish relevant standard operating procedures (SOPs). Moreover, investigators should receive training on SOPs to guarantee that all investigators fully understand and strictly follow the protocol before the trial. Improvement of scientific research ability and strengthening supervision are important measures to avoid potential bias and improve the quality of clinical trials in China.

When the investigators report the clinical trials, investigators should report in accordance with Consolidated Standards of Reporting Trials (CONSORT) or other guidelines related to clinical trial reporting. Reporting clinical trials in accordance with reporting guidelines could enable readers to understand a trial’s conduct and to assess the validity of its results [107]. Therefore, RCTs should be reported adhering to the CONSORT, observational studies should be reported referring to strengthening the reporting of observational studies in epidemiology (STROBE) [108], and the protocol of RCTs should comply with Standard Protocol Items: Recommendations for Interventional Trials (SPIRIT) [109]. Journals should pay more attention to the reporting quality of clinical trials and demand authors to obey to the reporting guidelines [62]. Thereby, the authors could report their findings in a transparent and standardized way, provide readers with accurate information regarding clinical trial processes, facilitate peer review, and enhance the quality of reporting. In addition, the guideline to strengthen the quality of clinical trials, or to establish evidence ecosystem can help the quality of clinical trials [110, 111].

### Limitations

There are several limitations of this systematic review. Firstly, due to the disciplines in most studies that were scattered, only thirteen studies could be divided into different disciplines. So, it is difficult to analyze the included studies by disciplines. Secondly, since all the included studies were secondary research, a certain bias may exist. Thirdly, among the included studies, the problems mainly focused on the RCTs, which may be not applicable to other types of clinical trials. Fourthly, the current study do not include study which is performed to assess quality of trials fousing in a specific disease or a type of study, so further studies are needed. Lastly, we included only Chinese language and English language papers, so the language bias may exist [112]. 

## Conclusions

In conclusion, the quality problems of clinical trials in China still exist, which includes problems related to the entire clinical trial process, including ethical review, registration, implementation, and reporting. Due to the limited quantity and quality of included studies, our conclusions need to be verified by high-quality studies.

## Supplementary Information

**Additional file 1: Supplementary material 1**: Completed PRISMA checklist.

**Additional file 2: Supplementary material 2**: All search strategy.

**Additional file 3: Supplementary material 3**: The full list of all 90 papers

## Data Availability

The datasets used and/or analyzed during the current study are available from the corresponding author on reasonable request.

## Abbreviations

CFDA: China Food and Drug Administration
GCP: Good Clinical Practice
PRISMA: Preferred Reporting Items and Systematic Reviews and Meta-Analysis
ICMJE: International Committee of Medical Journal Editors
RCTs: Randomized control trials
SOPs: Standard operating procedures
CONSORT: Consolidated Standards of Reporting Trials
STROBE: Strengthening the Reporting of Observational Studies in Epidemiology
