# Supplementary material for: Quality problems of clinical trials in China: evidence from quality related studies
Source: Trials. 2022 Apr 23;23:343. doi: 10.1186/s13063-022-06281-1 (PMC9034627; doi:10.1186/s13063-022-06281-1)
Supplement: Supplementary file 2 — Additional file 2: Supplementary material 2: All search strategy. [file 13063_2022_6281_MOESM2_ESM.docx]

**Supplementary material 2**

**China National Knowledge Infrastructure Search strategy**

（SU=质量 OR SU=问题 OR SU=实施 OR SU=评价） AND SU=临床试验 AND SU=中国**the Chinese Science and Technology Periodical Database Search strategy**

(M=质量 OR M=问题 OR M=实施 OR M=评价) AND M=临床试验 AND M=中国

**Wanfang database Search strategy**

(主题=质量 OR 主题=问题 OR 主题=实施 OR 主题=评价) AND 主题=临床试验 AND 主题=中国

**China Biology Medicine Search strategy**

("质量"[加权:扩展] OR "问题"[加权:扩展] OR "实施"[加权:扩展] OR "评价"[加权:扩展]) AND "临床试验"[加权:扩展] AND "中国"[加权:扩展]

**Pubmed Search strategy**

#1 quality

#2 status

#3 situation

#4 issue

#5 deficiency

#6 #1 OR #2 OR #3 OR #4 OR #5

#7 trial [Title/Abstract]

#8 China

#9 Chinese

#10 #8 OR #9

#11 #6 AND #7 AND #10

**Embase Search strategy**

#1 quality OR status OR situation OR issue OR deficiency

#2 trial

#3 China OR Chinese

#4 #1 AND #2 AND #3
