# Supplementary material for: Quality problems of clinical trials in China: evidence from quality related studies
Source: Trials. 2022 Apr 23;23:343. doi: 10.1186/s13063-022-06281-1 (PMC9034627; doi:10.1186/s13063-022-06281-1)
Supplement: Supplementary file 3 — Additional file 3: Supplementary material 3: The full list of all 90 papers [file 13063_2022_6281_MOESM3_ESM.docx]

**The list of include studies**

| Number | Title |
| --- | --- |
| 1 | Problem analysis and solution after ethical review in clinical trial [1]. |
| 2 | Survey of subjects’ understanding of informed consent in drug clinical trials [2]. |
| 3 | Wang Z. The necessity and feasibility of establishing technical specifications for registry of Chinese medicine clinical study [3]. |
| 4 | Analysis and reflection on registration of Chinese clinical research on COVID-19 [4]. |
| 5 | Perspectives on the implementation of ethics committee’s responsibilities from drug clinical trial data inspection [5]. |
| 6 | Analysis of registered drug clinical research of coronavirus disease 2019 [6]. |
| 7 | Problems and countermeasures in ethical review system of clinical research in China [7]. |
| 8 | The past 10 years of clinical trial registration in China: status and challenge [8]. |
| 9 | An investigation based on registered clinical trials on Chinese clinical trial registry for exploring the factors of impacting quality of clinical trials [9]. |
| 10 | Common problems and solutions in the design of TCM clinical trial research programs [10]. |
| 11 | Problems and countermeasures of ethical review and supervision of high-risk medical technologies: take gene editing technology as an example [11]. |
| 12 | Analysis of clinical trial registration information of COVID-19 in China [12]. |
| 13 | Study on the common problems of and countermeasures for the supervision of institutions for drug clinical trial [13]. |
| 14 | Problems and countermeasures of drug clinical trials in China [14]. |
| 15 | Ethics committee’s management of noncompliance/violation or protocol deviation report in clinical research [15]. |
| 16 | Discussion on the problems and suggestions in ethical review of clinical scientific research projects in China [16]. |
| 17 | Investigation and analysis of current status of ethical review of serious adverse events in clinical trials in Jiangsu Province [17]. |
| 18 | Common problems and countermeasures in the implementation of drug clinical trial project [18]. |
| 19 | Ethical problems and analysis of countermeasures in drug clinical trials [19]. |
| 20 | The existing problems in the construction and development of medical ethics committee [20]. |
| 21 | Key points of ethical review in drug clinical trials [21]. |
| 22 | Comparison and analysis of issues existing in informed consent forms of clinical trials sponsored by foreign and domestic enterprises [22]. |
| 23 | Problems and countermeasures for the ethical review of drug clinical trials [23]. |
| 24 | Research on problems and countermeasures of drug clinical trials [24]. |
| 25 | Research on problems and countermeasures of the management of clinical trials’ contracts in medical institutions [25]. |
| 26 | Problems and countermeasures of quality control of drug clinical trial items [26]. |
| 27 | Analysis of common problems and measures in the process of drug clinical trial [27]. |
| 28 | Problems and countermeasures of drug clinical trial SAE report [28]. |
| 29 | Analysis of adverse event records in quality control of drug clinical trial institutions [29]. |
| 30 | Analysis of common problems and measures in the process of drug clinical trial [30]. |
| 31 | Problems and countermeasures in the management of drug clinical trial coordinators [31]. |
| 32 | Thinking on the writing of informed consent for drug clinical trial [32]. |
| 33 | Managerial experience in clinical research process of Chinese medicine [33]. |
| 34 | Existing problems and solution of informed consent in clinical research [34]. |
| 35 | Investigation and analysis of existing problems in clinical trials of drugs and countermeasures [35]. |
| 36 | Discussion on the problems and countermeasures in the management of experimental drugs [36]. |
| 37 | Problems and countermeasures in contract management and fund management of drug clinical trial [37]. |
| 38 | Developing status of clinical research in China and the existing problems, strategies and practices of clinical research of traditional Chinese medicine [38]. |
| 39 | Common problems and countermeasures of clinical trial agreement [39]. |
| 40 | Problems discovered in drug clinical trial quality monitoring and countermeasures: including problems of post-marketing reevaluation of Chinese patent drugs [40]. |
| 41 | Analysis of quality management status and existing issues in the drug clinical trial institution in our hospital [41]. |
| 42 | Sources, responsibilities, problems and countermeasures of clinical research coordinators in clinical trials [42]. |
| 43 | Problems and countermeasures in the implementation of quality management standard for drug clinical trials in general hospitals [43]. |
| 44 | Analysis of regulatory issues of adverse events in drug clinical trials [44]. |
| 45 | Analysis of issues in drug clinical trials [45]. |
| 46 | Discussion on the issues and countermeasures in clinical trial insurance [46]. |
| 47 | Problems on the management of hospital drug clinical trial and analysis of measures [47]. |
| 48 | Analysis of problems in clinical informed consent [48]. |
| 49 | Problems and solutions of serious adverse event report in clinical research from perspective of ethics review [49]. |
| 50 | On problems and countermeasures of drug clinical trial data cleanup in China [50]. |
| 51 | Thoughts on the quality of clinical trial of new drugs in China [51]. |
| 52 | Reflections on informed consent in clinical trials [52]. |
| 53 | Problems and countermeasures of drug management in clinical trials [53]. |
| 54 | Clinical trial data quality problems and improvement measures [54]. |
| 55 | Problems and countermeasures for data verification of drug clinical trial [55]. |
| 56 | Methodological quality and reporting quality evaluation of randomized controlled trials published in China Journal of Chinese Materia Medica [56]. |
| 57 | Analyze the causes and control measures of common problems in clinical trials [57]. |
| 58 | Discussion on common problems and reporting standards of clinical trial protocols in China [58]. |
| 59 | The status of registrations, ethical reviews and informed consent forms in RCTs of high impact factor Chinese medical journals [59]. |
| 60 | Problems and countermeasures in the management of external clinical trial coordinators [60]. |
| 61 | Journal of Chengdu University of Traditional Chinese Medicine: literature review of randomized controlled clinical trials [61]. |
| 62 | Attention should be paid to the statistical problem of medical clinical trial papers -- the development of self-check list of statistical report items [62]. |
| 63 | Research on protection of participants’ rights and interests in clinical trials: a case study of a clinical trial agency of one 3A grade hospitals in Beijing [63]. |
| 64 | Informed consent problems and countermeasures in drug clinical trial [64]. |
| 65 | Quality evaluation of domestic literature reports on randomized controlled trial of acupuncture treatment of angina pectoris [65]. |
| 66 | Assessment of clinical studies published in Chinese Journal of Infectious Diseases for 18 years [66]. |
| 67 | Quality evaluation of randomized controlled clinical trials in Chinese Journal of Integrated Traditional and Western Medicine [67]. |
| 68 | Quality management of clinical trials in China [68]. |
| 69 | Analysis of data recording defects in case report form of TCM clinical trial [69]. |
| 70 | [Informed consent information integrity research [70].](https://doi.org/10.5428/pcar20110216.) |
| 71 | Clinical trial status and trends from quality management perspective [71]. |
| 72 | Perspectives on the implementation of sponsors’ responsibilities from the drug clinical trial data inspection [72]. |
| 73 | Perspectives on the implementation of responsibilities of drug clinical trial institutions from drug clinical trial data inspection [73]. |
| 74 | Perspectives on the performing of investigators’ responsibilities from drug clinical trial data inspection [74]. |
| 75 | Studies on current situation and strategies for subjects recruited of phase I clinical trial of drugs in China [75]. |
| 76 | Report quality assessment of randomized controlled trial abstracts from Chinese clinical journals [76]. |
| 77 | Analysis of common defects in the clinical trial reports of traditional Chinese medicines [77]. |
| 78 | Problem analysis and countermeasure of informed consent and ethical review of clinical study [78]. |
| 79 | Analysis of the problem of informed consent in clinical trial [79]. |
| 80 | Common problems and analysis of informed consent in drug clinical trials [80]. |
| 81 | Analysis of defects and recommendation for “three levels quality control” system of clinical trials [81]. |
| 82 | Reporting quality assessment of noninferiority and equivalence randomized controlled trials related to traditional Chinese medicine [82]. |
| 83 | Bian Z, Li Y, Moher D. Further improve the reporting quality of clinical research in China [83]. |
| 84 | Analysis on quality of literatures related to randomized controlled trials on acupoint massage [84]. |
| 85 | Evaluation of methodological quality in clinical trials of traditional Chinese medicine [85]. |
| 86 | Quality assessment of randomized controlled trials related to traditional Chinese medicine published in Journal of Traditional Chinese Medicine in 2018 [86]. |
| 87 | An assessment of methodological quality of multi-center randomized controlled trials of stroke treatments conducted in Chinese Mainland [87]. |
| 88 | Blinding assessment in randomized controlled clinical trials [88]. |
| 89 | Common problems and countermeasures in clinical trials of traditional Chinese medicine [89]. |
| 90 | Issues in the clinical evaluation of the efficacy of traditional Chinese medicine [90]. |

1. Tian L, Zhang H. Problem analysis and solution after ethical review in clinical trial. Chin J Clin Pharmacol. 2019;35(06):584–6.

2. Feng L. Survey of subjects’ understanding of informed consent in drug clinical trials. Chin J New Drugs. 2018;27(24):2911–5.

3. Lu P, Liao X, Xie Y, Wang Z. The necessity and feasibility of establishing technical specifications for registry of Chinese medicine clinical study. Chin J Integr Tradit Western Med. 2015;35(01):14–8.

1. He Q, Kuang J, Tao M, Lu Y, Xiao W, Gao T, et al. Analysis and reflection on registration of Chinese clinical research on COVID-19. Pharm Clin Chin Materia Medica. 2020;11(5):1–4.
2. Gao R, Tang J, Fang X, Lei M, Zhang Q. Perspectives on the implementation of ethics committee’s responsibilities from drug clinical trial data inspection. Chin J New Drugs. 2019;28(20):2513–7.
3. Zhao S, Zhang B, Chang X, Liu Y, Li Y, Li J. Analysis of registered drug clinical research of coronavirus disease 2019. Chin J Hosp Pharm. 2020;40(14):1499–504.
4. Chen S. Problems and countermeasures in ethical review system of clinical research in China. Soft Sci Health. 2019;33(11):66–70.
5. Wu T, Minawaer A, Hao Y, Kong X, Chen S, Li Y. The past 10 years of clinical trial registration in China: status and challenge. Chin J Evid Based Med. 2018;18(6):522–5.
6. Wu T, Minawaer A, Bian Z, Shang H, Yang L, Hao Y, et al. An investigation based on registered clinical trials on Chinese clinical trial registry for exploring the factors of impacting quality of clinical trials. Chin J Evid Based Med. 2018;18(06):526–31.
7. Shen W, Wu X, Wang G, Liu J. Common problems and solutions in the design of TCM clinical trial research programs. Acta Chin Med Pharmacol. 2015;43(04):1–4.
8. Geng W, Qin D, Dai M, Jiang Y. Problems and countermeasures of ethical review and supervision of high-risk medical technologies: take gene editing technology as an example. Chin Med Ethics. 2020;33(6):695–8.
9. Xiang Y, Zeng C, Huang Z, Wang X, Zhang Z, Yang G. Analysis of clinical trial registration information of COVID-19 in China. Chin J Clin Pharmacol Ther. 2020;25(2):135–40.
10. Ding Z, Cong L, Wu B. Study on the common problems of and countermeasures for the supervision of institutions for drug clinical trial. Chin Pharm Affairs. 2018;32(03):299–304.
11. Zhang L, Zhao S, Guo C. Problems and countermeasures of drug clinical trials in China. Chin J Clin Ration Drug Use. 2018;11(04):162–3.
12. Wu C, Cao G, Wu R, Zou H. Ethics committee’s management of noncompliance/violation or protocol deviation report in clinical research. Chin Med Ethics. 2018;31(03):328–31.
13. Liu D, Zeng S, Deng F, Zhou J. Discussion on the problems and suggestions in ethical review of clinical scientific research projects in China. Chin Med Ethics. 2018;31(07):829–32.
14. Liu Q. Investigation and analysis of current status of ethical review of serious adverse events in clinical trials in Jiangsu Province: NanJin Medical University; 2017.
15. Zhao T, Ma L, Wang H, Xiang P. Common problems and countermeasures in the implementation of drug clinical trial project. China Modern Doctor. 2017;55(8):146–8.
16. Zhao J, Zhang X, Shen L. Ethical problems and analysis of countermeasures in drug clinical trials. J Qiqihar Med Univ. 2016;37(21):2748–9.
17. Yan X, Liu Z, Chen Y, Wang H. The existing problems in the construction and development of medical ethics committee. Chin Remedies Clin. 2016;16(03):363–5.
18. Li Y. Key points of ethical review in drug clinical trials. China Health Care Nutr. 2016;26(16):136–7.
19. Bai C, Fan X, Ren P, Li N. Comparison and analysis of issues existing in informed consent forms of clinical trials sponsored by foreign and domestic enterprises. Chin J New Drugs. 2015;24(15):1750–3.
20. Wang H, Wang C, Li S. Problems and countermeasures for the ethical review of drug clinical trials. Chin Hospital Manage. 2015;35(12):75–6.
21. Chen Y, YanX, Wang H. Research on problems and countermeasures of drug clinical trials. Soft Sci Health. 2015;29(11):691–4.
22. Cao Y, Cao G, Zhang J, Wu C, Zhan H, Mao Y. Research on problems and countermeasures of the management of clinical trials’ contracts in medical institutions. Chin J New Drugs. 2019;28(24):2997–3000.
23. Li G, Min J, Wang Y, Zhao X, Xia Y. Problems and countermeasures of quality control of drug clinical trial items. Pharm Care Res. 2019;19(05):392–4.
24. Hou Y, Zhao Q. Analysis of common problems and measures in the process of drug clinical trial. Electron J Pract Clin Nurs Scie. 2019;4(38):123.
25. Jin H, Zhang X, Mo X, Cui Y, Zhang G. Problems and countermeasures of drug clinical trial SAE report. Chin Hosp Pharm J. 2017;37(15):1530–2.
26. Zeng L, Pan X. Analysis of adverse event records in quality control of drug clinical trial institutions. Chin J Pharmacovigilance. 2018;15(11):693–5.
27. Feng C, Jiang Y. Analysis of common problems and measures in the process of drug clinical trial. Nurs Integr Tradit Chin Western Medi. 2017;3(03):137–9.
28. Feng C, Jiang Y. Problems and countermeasures in the management of drug clinical trial coordinators. J Front Med. 2018;8(29):390–2.
29. Ceng H, Wang Q. Thinking on the writing of informed consent for drug clinical trial. J Med Inform. 2016;29(31):258–9.
30. Gao C, Chen X, Wang J, Li Y, Tian S, Zhu C. Managerial experience in clinical research process of Chinese medicine. Guiding J Tradit Chin Med Pharm. 2017;23(18):6–8.
31. Liu D, Zeng S, Deng F, Zhou J. Existing problems and solution of informed consent in clinical research. Chin Med Ethics. 2018;31(06):732–5.
32. Liu F, Deng G, Li X, Wang G. Investigation and analysis of existing problems in clinical trials of drugs and countermeasures. Chin J New Drugs. 2017;26(17):2059–63.
33. Liu F, Deng G, Li X, Wang G. Discussion on the problems and countermeasures in the management of experimental drugs. China Med Herald. 2017;14(20):128–31.
34. Fan H, Wang H, Zhang H. Problems and countermeasures in contract management and fund management of drug clinical trial. Chin J Med Sci Res Manag. 2018;31(03):232–5.
35. Hu J, Zhang X, Jiang Y, Sun Y, Li Y, Tian G, et al. Developing status of clinical research in China and the existing problems, strategies and practices of clinical research of traditional Chinese medicine. Modernization Tradit Chin Med Materia Medica World Sci Technol. 2018;20(08):1417–21.
36. Liu J, Mi F, Zhao W, Zhang T, Chu N, Cai C. Common problems and countermeasures of clinical trial agreement. Hosp Adm J Chin People's Liberation Army. 2018;25(02):135–6.
37. Li J, Shang H, Zhao C. Problems discovered in drug clinical trial quality monitoring and countermeasures: including problems of post-marketing reevaluation of Chinese patent drugs. Chin J New Drugs. 2019;28(18):2184–8.
38. Chen L, Zhang S. Analysis of quality management status and existing issues in the drug clinical trial institution in our hospital. China Health Ind. 2017;14(10):160–1.
39. Liu L, Zhou J. Sources, responsibilities, problems and countermeasures of clinical research coordinators in clinical trials. J Int Pharm Res. 2018;45(07):512–6.
40. Mijiti B, Mohemaiti P. Problems and countermeasures in the implementation of quality management standard for drug clinical trials in general hospitals. Xinjiang Med J. 2016;46(12):1590–2.
41. Peng P, Yuan W, Hu Y, Tang J, Jiang J. Analysis of regulatory issues of adverse events in drug clinical trials. Chin J Clin Pharmacol Ther. 2018;23(01):78–82.
42. Peng P, Yuan W, Hu Y, Tang J, Zhu L, Jiang J. Analysis of issues in drug clinical trials. Chin J New Drugs Clin Remedies. 2015;34(05):339–42.
43. Peng P, Yuan W, Hu Y, Tang J, Zhu L, He M, et al. Discussion on the issues and countermeasures in clinical trial insurance. Chin Med Ethics. 2017;30(03):328–30.
44. Chen Q, Liang D. Problems on the management of hospital drug clinical trial and analysis of measures. Capital Food Med. 2015;22(04):14.
45. Zhao S, Liu X, Fu Z, Jiang M. Analysis of problems in clinical informed consent. Chin J New Drugs. 2016;25(23):2692–5.
46. Li X, Zhang X, Liu S, Liang W, Liu J. Problems and solutions of serious adverse event report in clinical research from perspective of ethics review. Chin J New Drugs Clin Remedies. 2017;36(07):393–6.
47. Han X, Luo H, Zhang D. On problems and countermeasures of drug clinical trial data cleanup in China. Chin Pharm Affairs. 2018;32(07):853–7.
48. Hu Y, Tang J, Peng P, Yuan W. Thoughts on the quality of clinical trial of new drugs in China. J Guangdong Pharm Univ. 2019;35(02):279–84.
49. Xing X, Zhang J, Deng R, Wang X, Gao J. Reflections on informed consent in clinical trials. Electron J Clin Med Lit. 2019;6(82):188–9.
50. Liang Y, Lu J. Problems and countermeasures of drug management in clinical trials. J Pract Med Tech. 2015;22(04):440–2.
51. Huang A, Sun R, Wang Y, Li H, Xu K, Yang C, et al. Clinical trial data quality problems and improvement measures. J China Japan Friendship Hosp. 2015;29(04):224–7.
52. Peng Z, Wang C, Chen Y, Yang C, Xu W, Xu X. Problems and countermeasures for data verification of drug clinical trial. Chin J Hosp Pharm. 2018;38(21):2267–72.
53. Yu D, Xie Y, Liao X, Zhi Y, Jiang J, Chen W. Methodological quality and reporting quality evaluation of randomized controlled trials published in China Journal of Chinese Materia Medica. China J Chin Materia Medica. 2018;43(04):833–9.
54. He G, Zeng T, Zhang W, Zhang L, Zhang X, Xu C. Analyze the causes and control measures of common problems in clinical trials. Chin J New Drugs Clin Remedies. 2018;37(01):24–8.
55. Huang H, Shi Y, Qiu Y, Zhang S, Lu D, Li L, et al. Discussion on common problems and reporting standards of clinical trial protocols in China. Chin J Med Sci Res Manag. 2018;31(03):161–6.
56. Zhao H, Zhang J, Guo L, Yang F, Zhang M, Li Y, et al. The status of registrations, ethical reviews and informed consent forms in RCTs of high impact factor Chinese medical journals. Chin J Evid Based Med. 2018;18(07):735–9.
57. Li D, Jiang W. Problems and countermeasures in the management of external clinical trial coordinators. Chin J Modern Nurs. 2015;21(31):3834–5.
58. Chi H, Mao B, Wang L, Zhang Y. Journal of Chengdu University of Traditional Chinese Medicine: literature review of randomized controlled clinical trials. West China Med J. 2000;15(03):272–3.
59. Liu Q, Fang J. Attention should be paid to the statistical problem of medical clinical trial papers -- the development of self-check list of statistical report items. Natl Med J China. 2007;87(34):2446–8.
60. Zheng J, Li Y. Research on protection of participants’ rights and interests in clinical trials: a case study of a clinical trial agency of one 3A grade hospitals in Beijing. Chin J New Drug. 2015;24(15):1754–9.
61. Ji L, Zhao Q. Informed consent problems and countermeasures in drug clinical trial. Chin Pharm Affairs. 2015;29(04):412–6.
62. Chen L, Yao J, Chen J, Zhao L, Liang F. Quality evaluation of domestic literature reports on randomized controlled trial of acupuncture treatment of angina pectoris. Modernization Tradit Chin Med Materia Medica World Sci Technol. 2020;8:2710–6.
63. Wei M, Yang M, Zhang M, Wang W, Liu J. Assessment of clinical studies published in Chinese Journal of Infectious Diseases for 18 years. Chin J Evid Based Med. 2001;1(04):233–4.
64. Zhang M, Yang F, Li Y, Liu Y, Zhao M, Zheng W, et al. Quality evaluation of randomized controlled clinical trials in Chinese Journal of Integrated Traditional and Western Medicine. Chin J Evid Based Med. 2017;17(03):357–63.
65. Ji P. Quality management of clinical trials in China. Chin J New Drugs. 2013;22(1):13–6.
66. Liu Q, Wang Y, Chang T. Analysis of data recording defects in case report form of TCM clinical trial. Chin J Inf Tradit Chin Med. 2013;20(02):4–5.
67. Huang J, Shen Na, Liu H, Pu J, Hu J, Xiang Y. Informed consent information integrity research. Pharm Care Res. 2011;11(02):123–6. <https://doi.org/10.5428/pcar20110216.>
68. Li Q, Chen J, Zhang P, Zhou L. Clinical trial status and trends from quality management perspective. Chin J New Drugs. 2014;23(8):871–8.
69. Gao R, Wang A, Tang J, Fang X, Wang J. Perspectives on the implementation of sponsors’ responsibilities from the drug clinical trial data inspection. Chin J New Drugs. 2019;28(8):973–7.
70. Gao R, Ning J, Wang A, Fang X, Zhang R. Perspectives on the implementation of responsibilities of drug clinical trial institutions from drug clinical trial data inspection. Chin J New Drugs. 2019;28(20):2518–23.
71. Gao R, Lv S, Li X, Lei M, Qian X. Perspectives on the performing of investigators’ responsibilities from drug clinical trial data inspection. Chin J New Drugs. 2019;28(20):2508–12.
72. Han S, Kong Y, Sheng X, Zhao X, Zhou Y, Cui Y. Studies on current situation and strategies for subjects recruited of phase I clinical trial of drugs in China. Chin J Clin Pharmacol. 2016;32(18):1722–5.
73. Shang S, Yan Y, Wu X. Report quality assessment of randomized controlled trial abstracts from Chinese clinical journals. Chin J Sci Tech Periodicals. 2017;28(12):1121–7.
74. Pei X. Analysis of common defects in the clinical trial reports of traditional Chinese medicines. Chin J New Drugs. 2009;18(15):1391–3.
75. Luo X, Ma X, Wang Y, Duan J. Problem analysis and countermeasure of informed consent and ethical review of clinical study. China Modern Med. 2016;23(8):176–9.
76. Wu X, Wang L, Liu M, Yang X, Peng L, Zhang Y, et al. Analysis of the problem of informed consent in clinical trial. Chin Med Ethics. 2018;31(01):20–3.
77. Yan H. Common problems and analysis of informed consent in drug clinical trials. Medical Community. 2018;23:1.
78. Shen Y, Zhang Z, Zhang Q, Li C, Wang B. Analysis of defects and recommendation for “three levels quality control” system of clinical trials. Chin J New Drugs Clin Remedies. 2016;35(10):721–3.
79. Liu Y, Liang W. Reporting quality assessment of noninferiority and equivalence randomized controlled trials related to traditional Chinese medicine. Chin J Evid Based Med. 2011;11(03):336–40.
80. Bian Z, Li Y, Moher D. Further improve the reporting quality of clinical research in China. Chin J Evid Based Med. 2020;20(12):1365–6.
81. Xiang C, Zhang W, Chen X, Hu Hong, Wen J, Zhao C, et al. Analysis on quality of literatures related to randomized controlled trials on acupoint massage. Chin Nurs Res. 2020;34(12):2228–30.
82. Cui Z, Bian Y. Evaluation of methodological quality in clinical trials of traditional Chinese medicine. Lishizhen Med Materia Medica Res. 2019;30(09):2302–4.
83. Long D, Liu Z, Li X, Liu H. Quality assessment of randomized controlled trials related to traditional Chinese medicine published in Journal of Traditional Chinese Medicine in 2018. Chin J Integr Med Cardio Cerebrovasc Dis. 2021;19(11):1935–9.
84. He S, Zhang T, Sun W, Qiu Z. An assessment of methodological quality of multi-center randomized controlled trials of stroke treatments conducted in Chinese Mainland. West China Med J. 2020;35(6):673–8.
85. Yan S, He L, Liu B. Blinding assessment in randomized controlled clinical trials. Chin J Evid Based Med. 2014;14(05):631–5.
86. Li T, Wang G, Wang L. Common problems and countermeasures in clinical trials of traditional Chinese medicine. Chin J Integr Tradit West Med. 2006;26(04):298–302.
87. Gao X, Ma Y, Zeng Y, Li G. Issues in the clinical evaluation of the efficacy of traditional Chinese medicine. Liaoning J Tradit Chin Med. 2015;42(07):1217–9.
